# Nutritional status and Cardiometabolic health among adolescents; findings from southwestern Nigeria

**DOI:** 10.1186/s40795-019-0308-5

**Published:** 2019-12-02

**Authors:** Adeleye Abiodun Adeomi, Iyanuoluwa Odunayo Adelusi, Praise Oluwatooni Adedeji, Adedoyin Esther Awofeso, Olajumoke Omotoyosi Oroleye, Doyinfunmi Lydia Gbadegesin

**Affiliations:** Community Health Department, Obafemi Awolowo University, College of Health Sciences, Ile-Ife, Osun State Nigeria

**Keywords:** Nutritional status, Overweight, Obesity, Cardio-metabolic, Hypertension, Adolescents

## Abstract

**Background:**

Obesity has been associated with an increased risk for cardio-metabolic diseases. The prevalence of obesity among adolescents is increasing worldwide, including Nigeria, but only little data exist on the relationship of nutritional status with cardio-metabolic health among adolescents in Nigeria. This study therefore investigated the relationship of nutrition status, raised blood pressure, glucose intolerance and risk for metabolic diseases among adolescents in southwestern Nigeria.

**Methods:**

This was a cross-sectional study conducted among 313 in-school adolescents in Ile-Ife, southwestern Nigeria. The respondents were selected using multi-stage sampling technique, and data were collected using pre-tested structured questionnaires. Anthropometric, blood pressure and random blood glucose measurements were done using standard protocols. Nutritional assessment was done using the World Health Organization (WHO) 2007 reference. Pre-hypertension and hypertension were measured using percentiles for gender, age and height. Analysis was done using IBM SPSS and the level of significance was set at *p* ≤ 0.05.

**Results:**

The mean age of the respondents was 14.4 ± 2.0 years. The prevalence of overweight and obesity was 10.2%, and it was significantly higher among females (12.5%) than males (7.0%). The prevalence of systolic and diastolic pre-hypertension was 10.9 and 11.5% respectively, while the prevalence for systolic and diastolic hypertension were 14.4 and 8.6% respectively. Using WHtR to assess cardio-metabolic risk, 7.3% of the respondents were at risk. There were statistically significant relationships between BMI, WHtR and blood pressure levels (< 0.05).

**Conclusions:**

The prevalence of overweight/obesity among the adolescents was relatively high, and this was significantly associated with elevated blood pressure and increased risk for cardio-metabolic diseases. There is need for more interest and intervention by stakeholders into the cardio-metabolic health of adolescents in Nigeria.

**Electronic supplementary material:**

The online version of this article (10.1186/s40795-019-0308-5) contains supplementary material, which is available to authorized users.

## Background

The prevalence of overweight and obesity among children and adolescents is rising all over the world [1]. The global burden of diseases project found that the prevalence of overweight/obesity among adolescents nearly doubled from 1990 to 2016, with a current prevalence of 18% [2]. It is more disturbing that this rise in the prevalence of overweight and obesity is faster in low and middle income countries (LMIC) compared to the developed countries [3]. There are studies in Africa which are already reporting prevalence of overweight/obesity higher than this global average [4, 5].

The increasing prevalence of overweight and obesity have been attributed to nutrition transition [6]. According to Popkin [7], nutrition transition depicts the change from the traditional food patterns rich in fruits, vegetables and fibre, to what has been called the western food pattern. This western food pattern describes food rich in sugar, refined foods, saturated fats with little or low fibre. Nutrition transition also involves the reduction in energy expenditure and the more sedentary lifestyles brought about by technological improvements.

Nigeria is experiencing nutrition transition, with rapid urbanization and associated adoption of western diets and reducing energy expenditure among children and adolescents [8]. In the past few decades, studies on the nutritional status of children hardly reported childhood overweight or obesity [9], but this is changing in recent times. Some Nigerian studies are reporting prevalence of adolescent overweight/obesity close to the global average [10, 11].

The major challenge with the increasing prevalence of overweight/obesity among adolescents is the morbidity and mortality that has been associated with these nutritional states [12]. Overweight/obesity among children and adolescents has been associated with an increased risk for cardiovascular and metabolic diseases, which have been termed cardio-metabolic diseases [13, 14]. Increasing number of studies are reporting significant relationships between rising body mass index and high blood pressure, glucose intolerance and metabolic risks.

There are only few studies that have investigated the relationship between the nutritional status and the blood pressure levels of adolescents in Nigeria [15, 16]. No study however was found in the study location that had investigated the relationship of nutritional status with glucose levels and risk for metabolic diseases (cardio-metabolic health). This study therefore investigated the relationship of nutrition status, raised blood pressure, glucose intolerance and risk for metabolic diseases among adolescents in southwestern Nigeria.

## Materials and methods

### Study location and study population

The study was carried out in Ile-Ife, which is an ancient city in Osun State, Southwestern Nigeria. The respondents were adolescents aged 10 to 19 years attending secondary schools in public and private schools in Ile-Ife. Adolescents who were acutely ill or had chronic illnesses like sickle cell disease anaemia that could affect their weight were excluded. Others with disabilities that made them unable to stand were also excluded.

### Sample size and sampling technique

The sample size was calculated to get an absolute precision of ±5% using STATCALC on the Epi-Info software. The proportion of expected outcome was taken as 22.2% which was the proportion of in-school adolescents with pre-hypertension in a similar study carried out in eastern Nigeria [16], with an acceptable margin of error of 5%. A total of 313 adolescents were recruited from 4 secondary schools in Ile-Ife using multi-stage sampling technique.

### Research instruments and data collection methods

A self-developed, pre-tested structured questionnaire (Additional file 1) was used for data collection using the assisted self-administered method. Assessment of the socio-demographic characteristics of the adolescents, risky behaviour and self-reported history of hypertension or diabetes mellitus among the parents was done. Height was measured to the nearest 0.1 m using the stadiometer (Leceister® Height Measure, Seca, UK), weight was measured using the Seca® electronic bathroom weighing scale (SECA GmbH & Co, Germany) and waist and hip circumferences using the Goldfish brand non-elastic tape measure. The anthropometric measurements were done according to standard protocols recommended by the International Society for the Advancement of Kinanthropometry [17].

Blood pressure was measured using the OMRON 2 digital sphygmomanometer and random blood sugar levels using OnCall Plus blood glucose meter. Blood pressure (systolic and diastolic blood pressure) was measured with the student in a sitting position, back supported, feet flat on the floor, clothing sleeve rolled back to leave upper arm uncovered, right arm supported and cubital fossa (inner elbow) approximately at heart level, having sat for at least 5 to 10 min to rest before measurement. Two measurements were taken 10 min apart, and the average was taken as the blood pressure. For the blood sugar test, single-use disposable non sterile gloves were used for each participant, as well as single-use disposable lancet device. Tests were carried out as per manufacturer’s instructions. It was challenging to secure the cooperation of the adolescents to fast overnight, so the random blood sugar level was measured in millimole per litre (mmol/L). Respondents were classified such that those with blood glucose level less than 7.8 were normal, those with blood glucose level 7.8 to 11.0 had impaired glucose tolerance, greater than 11.0 had diabetes mellitus [18].

### Measurement of outcome variables

Pre-hypertension was taken as systolic blood pressure (SBP) and diastolic blood pressure (DBP) ≥ 90th percentile, but <95th percentile for gender, age and height, while hypertension was taken as SBP and DBP ≥ 95th percentile for gender, age and height [19].

Nutritional status of the respondents were determined using the WHO 2007 reference for children 5–19 years. Body Mass index (BMI) was calculated by dividing the weight in kilograms by the height in meter^2^. BMI-for-age Z-scores from the WHO reference charts were then used to classify the respondents as underweight (<− 2 SD), normal (≥ − 2 to ≤ + 1), overweight (> + 1 to + 2) and obese (> + 2). Waist hip ratio (WHR) was calculated as waist circumference in centimeters (cm) divided by hip circumference in cm. WHR was classified such that males with ratio equal to or greater than 0.9 and females with ratio equal to or greater than 0.85 were abnormal. Risk for metabolic diseases was assessed using waist-to-height (WHtR) ratio, which has been shown to be the best predictor of cardiovascular risk and mortality [12]. WHtR was calculated by dividing waist circumference in cm by height in cm, and those with ≥0.5 were classified as high.

### Data analysis

The questionnaires were manually sorted out, entered into a computer and the obtained data was analyzed using IBM SPSS version 20. Descriptive analysis of all the variables measured were first done, and the categorical variables were reported as frequencies and proportions/percentages, while the continuous variables were reported as means ± standard deviation. Cross-tabulations were done to test for associations between the different categorical variables (in line with the objective of the study) using the chi-square test. Fisher’s exact test was used when there was an expected value was less than 5. Correlation analysis was done to test the strength and direction of associations between the BMI, SBP, DBP, WHR and WHtR as continuous variables. Binary logistic regression was done to identify the factors associated with elevated blood pressure. The age, sex and other factors that were significantly associated with elevated blood pressure at the bivariate analysis level were entered into the model. Logistics regression was not done for elevated blood sugar level because no factors were significantly associated at bivariate level. Level of significance was set at *p* < 0.05 for this study.

### Ethical considerations

Ethical clearance was obtained from the Ethical Review Committee, Institute of Public Health, Obafemi Awolowo University, Ile-Ife. Permission was obtained from the heads of the selected schools. The participants’ information sheet and consent was given to the parents and the adolescents who were 18 years or above. Important information on the participants’ information sheet included that the information volunteered will be kept confidential as all questionnaires were coded without names or addresses of respondents. It also emphasized that participants were free to opt-out if they were not comfortable with the information in the questionnaire. Signed consent forms were then obtained from the parents and adolescents who are 18 years and above, while assent was obtained from adolescents who were less than 18 years.

## Results

The mean age of the respondents was 14.4 ± 2.0 years, more of them were females (58.5%) and 243 (77.6%) lived with both parents. Most of the respondents were from monogamamous family settings (84.0%) with an average of 3.9 ± 1.6 children in their families. Concerning the occupation of the parents, 55.0 and 31.0% of the fathers were skilled (professionals) and semi-skilled (artisans) respectively, while 40.9 and 48.9% of the mothers were skilled and semi-skilled respectively. Thirteen (4.2%) of the respondents had ever smoked, but only 4 (1.3%) of them were current smokers. There was a history of taking alcoholic beverages among 65 (20.8%) of them, but 22 (7.0%) were currently taking alcoholic beverages. Only few of the respondents reported the history of hypertension and diabetes mellitus in their fathers (3.8, 2.9%) and mothers (4.5, 1.6%) respectively, while many of them did not know.

The age and sex distribution of overweight and obesity are shown on Table 1. Overall, 10.2% of the adolescents were overweight and obese (males 7.0%, females 12.5%). When association between the nutritional status of the adolescents was tested with their socio-demographic characteristics, gender (overweight/obese males Vs females was 9 Vs 23; *p* = 0.036) and the mothers’ occupation (overweight/obese among the four categories of mother’s occupation were 4, 18, 10 and 0; *p* = 0.008) had statistically significant associations. This relationship was such that females and children of skilled mothers (professionals) were significantly more likely to be overweight/obese. Using random blood sugar (RBS), 6 (3.4%) of the respondents had glucose intolerance but none was in the diabetic range. Cross tabulations of the RBS categories with potential explanatory variables yielded no statistically significant associations.

**Table 1:** Age and sex distribution of under- and over-nutrition among respondents

Age	Males (%)				Females (%)				Total (%)				
	N	Uw	Ow	Ob	N	Uw	Ow	Ob	N	Uw	Ow	Ob	Ow/Ob
10	5	0.0	20.0	0.0	5	0.0	20.0	0.0	10	0.0	20.0	0.0	20.0
11	7	0.0	0.0	0.0	11	18.2	9.1	0.0	18	11.1	5.6	0.0	5.6
12	9	44.4	0.0	0.0	10	0.0	10.0	0.0	19	21.1	5.3	0.0	5.3
13	10	20.0	10.0	0.0	31	9.7	16.1	3.2	41	12.2	14.6	2.4	17.0
14	26	15.4	23.1	0.0	57	12.3	10.5	1.8	83	13.3	14.5	1.2	15.7
15	21	19.0	0.0	0.0	30	10.0	10.0	0.0	51	13.7	5.9	0.0	5.9
16	14	21.4	0.0	7.1	25	0.0	4.0	4.0	39	7.7	2.6	5.1	7.7
17	24	4.2	0.0	0.0	10	10.0	10.0	0.0	34	5.9	2.9	0.0	2.9
18	10	10.0	0.0	0.0	4	0.0	25.0	0.0	14	7.1	7.1	0.0	7.1
19	4	75.0	0.0	0.0	0	0.0	0.0	0.0	4	75.0	0.0	0.0	0.0
Total	130	16.9	6.2	0.8	183	8.7	10.9	1.6	313	12.1	8.9	1.3	10.2

*Uw* Underweight, *Ow* Overweight, *Ob* Obesity

The prevalence of systolic and diastolic pre-hypertension was 10.9 and 11.5% respectively, while the prevalence for systolic and diastolic hypertension were 14.4 and 8.6% respectively. (Table 2). Table 3 shows the sex distribution of WHR, WHtR, BP and RBS, with statistically significant gender differences in WHR, WHtR and BP categories. The independent factors that were significantly associated with elevated blood pressure are as show in Table 4. When these were entered into a binary logistic regression model, gender, current alcohol intake, BMI and WHtR categories showed statistically significant associations (*p* < 0.05). Current alcohol drinkers were 3 times (Odds ratio/Confidence interval [OR/CI] = 2.95/1.14–7.63; *p* = 0.025) more likely than non-drinkers, females were 2 times more likely than males (OR/CI = 1.88/1.03–3.14; *p* = 0.036), overweight/obese adolescents were 6 times (OR/CI = 5.91/1.98–20.34; *p* = 0.005) than those underweight and those with high WHtR were 2 times (OR/CI = 2.05/0.36–6.01; *p* = 0.013) more likely than those with low WHtR to have elevated blood pressure.

**Table 2:** Age distribution of Pre-hypertension and hypertension among respondents

Age	Systolic (%)				Diastolic (%)				Combined EBP
	N	pHT	HT	ESBP		pHT	HT	EDBP	
10	10	10.0	0.0	10.0		10.0	0.0	10.0	10.0
11	18	16.7	5.6	22.2		5.6	16.7	22.2	27.8
12	19	15.8	5.3	21.1		10.5	0.0	10.5	26.3
13	41	2.4	22.0	24.4		17.1	14.6	31.7	41.5
14	83	10.8	20.5	31.3		9.6	9.6	19.3	37.3
15	51	5.9	17.6	23.5		5.9	11.8	17.6	29.4
16	39	5.1	10.3	15.4		5.1	10.3	15.4	20.5
17	34	8.8	8.8	17.6		8.8	0.0	8.8	26.5
18	14	50.0	7.1	57.1		42.9	0.0	42.9	64.3
19	4	50.0	0.0	50.0		75.0	0.0	75.0	75.0
Total	313	10.9	14.4	25.2		11.5	8.6	20.1	32.9

*pHT* pre-hypertension, *HT* hypertension, *EBP* elevated blood pressure; combined EBP – number of respondents with systolic and/or diastolic EBP

**Table 3:** Sex distribution of WHR, Waist categories, WHtR, BP and RBS among Respondents

Variable	Sex distribution		Total (%)	Statistics
	Males (%)	Females (%)		
WHR				χ^2^ = 23.81^c^
Normal	118 (90.8)	123 (67.2)	241 (77.0)	df = 1
Abnormal	12 (9.2)	60 (32.8)	72 (23.0)	p < 0.001^a^
WHtR				χ^2^ = 4.01^c^
Low	125 (96.2)	165 (90.2)	290 (92.7)	df = 1
High	5 (3.8)	18 (9.8)	23 (7.3)	p = 0.045^a^
BP				χ^2^ = 4.59 ^c^
Normal	96 (73.8)	114 (62.3)	210 (67.1)	df = 1
Elevated	34 (26.2)	69 (37.7)	103 (32.9)	*p* = 0.032^a^
^b^RBS (*n* = 179)				χ^2^ = 0.344 ^c^
Normal	66 (95.7)	107 (97.3)	173 (96.6)	df = 1
Elevated	3 (4.3)	3 (2.7)	6 (3.4)	*p* = 0.558

*WHR* waist-hip ratio, *WHtR* waist-to-height ratio, *BP* blood pressure, *RBS* random blood sugar

^a^ statistically significant; ^b^ Only 179 adolescents consented to the test ^c^ Chi-square test used

**Table 4:** Factors associated with the Blood Pressure of the Respondents

Variable	Blood Pressure		Total (%)	Statistics
	Normal (%)	Elevated (%)		
Currently smoking				χ^2^ = 1.8^d^
Yes	1 (0.5)	3 (75.0)	4 (1.3)	df = 1
No	209 (99.5)	100 (97.1)	309 (98.)	*p* = 0.106
Currently taking alcoholic beverages				χ^2^ = 5.018^c^
Yes	10 (4.8)	12 (11.7)	22 (7.0)	df = 1
No	200 (95.2)	91 (88.3)	291 (93.0)	p = 0.025 ^a^
WHR				χ^2^ = 0.893^c^
Normal	165 (78.6)	76 (73.8)	241 (77.0)	df = 1
Abnormal	45 (21.4)	27 (26.2)	72 (23.0)	*p* = 0.391
WHtR				χ^2^ = 15.109^c^
Low	203 (96.7)	87 (84.5)	290 (92.7)	df = 1
High	7 (3.3)	16 (15.5)	23 (7.3)	p = 0.001^a^
BMI				
Underweight	29 (13.8)	9 (8.7)	38 (12.1)	χ^2^ = 24.971^c^
Normal	172 (81.9)	71 (68.9)	243 (77.6)	df = 2
Overweight/obese	9 (4.3)	23 (22.3)	32 (10.2)	p < 0.001^a^
^b^RBS (n = 179)				χ^2^ = 0.044^d^
Normal	108 (96.4)	65 (97.0)	173 (96.6)	df = 1
Elevated	4 (3.6)	2 (3.0)	6 (3.4)	*p* = 0.832

WHR – waist-hip ratio; WHtR – waist-to-height ratio; BMI – body mass index; RBS – random blood sugar

^a^ statistically significant; ^b^ only 179 (57.2%) of the respondents consented to the test; ^c^ Chi-square-test used; ^d^ Fisher’s exact test used

There was the risk for metabolic diseases as measured by the WHtR among 23 (7.3%) of the respondents. Gender (risk in males Vs females was 5 Vs 18; *p* = 0.045) was the only statistically significant explanatory variable among the socio-demographic characteristics. WHtR had a positive correlation with both systolic blood pressure (Correlation coefficient was 0.181; *p* = 0.001) and diastolic blood pressure (Correlation coefficient was 0.250; *p* < 0.001) and BMI (Correlation coefficient was 0.617; *p* < 0.001) (Table 5). The relationship between nutritional status of the respondents as measured using BMI, and the cardio-metabolic indicators is shown on Table 6. The blood pressure (elevated BP in normal weight Vs overweight/obese is 29.2% Vs 71.9%; p < 0.001) and the WHtR (high WHtR in normal weight Vs overweight/obese is 2.9% Vs 50%; p < 0.001) were significantly associated with the nutritional status of the adolescents. Although the prevalence of abnormal WHR was highest among the overweight/obese and lowest among the underweight, this difference was not statistically significant.

**Table 5:** Correlation analysis between nutritional and cardiometabolic indicators

	Correlation coefficient (*p*-value)		
	BMI	SBP	DBP
BMI	–	0.388 (< 0.001)^a^	0.255 (< 0.001)^a^
WHR	0.009 (0.873)	0.013 (0.816)	0.004 (0.950)
WHtR	0.617 (< 0.001)^a^	0.181 (0.001)^a^	0.250 (< 0.001)^a^

*WHR* waist-hip ratio, *WHtR* waist-to-height ratio; *BMI* body mass index, *SBP* – systolic blood pressure; *DBP* diastolic blood pressure ^a^ statistically significant

**Table 6:** Relationship between nutritional status and cardiometabolic indicators

Nutritional status (BMI)	N	Blood pressure		WHtR		WHR		^b^RBS	
		Normal	Elevated	Low	High	Normal	Ab-normal	Normal	Elevated
Underweight	38	76.3	23.7	100.0	0.0	81.6	18.4	94.1	5.9
Normal	243	70.8	29.2	97.1	2.9	77.4	22.6	96.3	3.7
Overweight/ Obese	32	28.1	71.9	50.0	50.0	68.8	31.2	100.0	0.0
Total	313	67.1	32.9	92.7	7.3	77.0	23.0	96.6	3.4
*p*-value		^c^ < 0.001^a^		^d^ < 0.001^a^		^c^0.428		^d^0.351	

WHR – waist-hip ratio; WHtR – waist-to-height ratio; BMI – body mass index; RBS – random blood sugar

^a^ statistically significant; ^b^ only 179 (57.2%) of the respondents consented to the test; ^c^ Chi-square-test used; ^d^ Fisher’s exact test used

## Discussion

The overall prevalence of overweight/obesity among the adolescents was 10.2%. This prevalence is higher than the country specific estimate, the regional estimate for West Africa and the global estimate by the joint child malnutrition estimates by WHO and UNICEF [1]. This prevalence is however similar to the 13.85% reported by the global burden of disease project [2]. Disparities also exist among studies carried out in Nigeria. While few of the recent studies in Nigeria have reported low prevalence of overweight/obesity [20, 21], more of the recent studies are reporting rates similar or even higher than the 10.2% found in this study [10, 11, 15, 22, 23]. Having a national perspective on the prevalence of obesity in Nigeria has been challenging, especially because different studies have used differing methodologies and reference values [24]. It can however be agreed that the prevalence of overweight/obesity is rising among adolescents in Nigeria. The overall prevalence of underweight in this same adolescent population was 12.1%. This typifies the double burden of disease, which is the paradoxical co-existence of under- and over-nutrition, which in this case is occurring at the population level [3, 25].

The significantly associated independent factors for the nutritional status of the respondents were gender and maternal occupation, such that females and children of professional mothers were more likely to be overweight/obese compared to the others. The association of gender, especially the female, has been similarly reported by previous similar studies. The effect of socio-economic and wealth status of the family and childhood obesity has been well documented. The finding in this study further goes to show that the socio-economic or wealth status of the mother may be what is really responsible. This may be understandable because of the usual bond/closeness of mothers and their children. Although there was marked difference in the prevalence of nutritional status across the different ages, with 20% at 10 years, 17% at 13 and 0% at 19%, these differences were however not statistically significant as was reported by other studies. This may be due to the number of respondents recruited in the different ages.

The low prevalence of glucose intolerance may be the reason why it did not show significant associations. It may not also be unrelated with the relatively small sample size of those who consented to take the test. Another possible reason may be a slower or more prolonged pathophysiology of glucose intolerance.

The prevalence of systolic and diastolic pre-hypertension was 10.9 and 11.5% respectively, while the prevalence for systolic and diastolic hypertension were 14.4 and 8.6% respectively. Overall, 32.9% had one form of elevated blood pressure or another. Similar findings, but with higher prevalence of pre-hypertension have been reported by Ejike et al. [16] in Nigeria, and Nkeh-Chungag et al. [26] in South Africa who reported (23.6 and 11.05%) and (21.2 and 12.3%) respectively, for prevalence of pre-hypertension and hypertension. A much lower prevalence was reported by Omisore et al. [15] in a similar study also carried out in southwestern Nigeria. This may however be due to the fact that they used a different methodology for classifying pre-hypertension and hypertension among the adolescents. This rising prevalence of elevated blood pressure should be a thing of concern among stakeholders for adolescent health in Nigeria. This finding corroborates the report of the global disease burden where the prevalence of non-communicable diseases among adolescents was put at 7.7%, similar to what was reported in some wealthy countries like United States of America, Spain and China [2]. The predictors of elevated blood pressure among the adolescents were female gender, alcohol intake, overweight/obesity and risk for metabolic disease as measured by WHtR.

The risk for cardio-metabolic diseases was 7.3%, as assessed by WHtR which has been found to be the best predictor of cardio-metabolic risk and mortality [12, 27], and has been used by similar studies [28]. To the best of our knowledge, this is the first study that assessed cardio-metabolic risk among adolescents using WHtR in Nigeria, hence no comparisons could be made within Nigeria. The proportion of adolescents with cardio-metabolic risk using WHtR in this study is low compared to the proportion in a large sample study in China. This difference is understandable since the two countries are socio-economically different, and the particular study reported overweight/obesity rates more than twice the prevalence found in this study. However, the findings of this study similarly show associations between indicators of nutritional status (BMI), WHtR and elevated blood pressure [29]. BMI and WHtR had significant positive correlation with SBP and DBP, and WHtR had the strongest, significant positive correlation with BMI. The proportion of those with elevated BP among overweight/obese were more than double the proportion among those with normal BMI. The proportion with high WHtR increased nearly 16 times among those with normal BMI to those overweight/obese. Another graphic representation of this relationship was the fact that half of those overweight/obese had high WHtR, while none of those underweight had high WHtR. These findings corroborates the need for monitoring of the nutritional status and cardio-metabolic risk or diseases even among adolescents [16].

The limitations of this study may include the fact that the respondents in this study were secondary school-attending adolescents in an urban community in southwestern Nigeria, generalization should therefore be done cautiously. There were only 4 respondents for age 19 and only 14 for age 18, because many of the age 18 and 19 would have left secondary school. There were relatively fewer respondents for the younger ages because of poor cooperation from them. Only 179 of the respondents consented to random glucose check, mainly for fear of needle prick, and majority did not agree to an overnight fast for fasting blood sugar.

Measurement of BP using oscilliometric devices and also on a single visit may tend to over-estimate the prevalence, and it may not be appropriate for diagnostic purposes. However, it was appropriate for the objectives of this study which was not for diagnostic nor individual interpretation rather, it was more like a screening at population level. Hence, the terms elevated blood pressure were the prominent terminology.

## Conclusion

The prevalence of overweight/obesity was relatively high, higher than WHO/UNICEF estimate for Nigeria. The prevalence of elevated blood pressure was high, and significantly more among females and those overweight/obese. The risk of cardio-metabolic diseases measured using WHtR was also relatively high, and significantly more among the overweight/obese. The nutritional status of the respondents was significantly associated with elevated blood pressure and increased risk for cardio-metabolic diseases There is need for more interest and intervention by stakeholders into the cardio-metabolic health of adolescents in Nigeria. More nationally representative data is needed on the nutritional status and cardio-metabolic diseases among Nigerian adolescents.

## Additional file

Additional file 1: Questionnaire. (DOCX 20 kb)

## Data Availability

The datasets used and/or analyzed during the current study are available from the corresponding author on reasonable request.

## Abbreviations

BMI: Body mass index
BP: Blood pressure
CI: Confidence interval
DBP: Diastolic blood pressure
LMIC: Low and middle income countries
OR: Odds ratio
RBS: Random blood sugar
SBP: Systolic blood pressure
SPSS: Statistical Package and service solutions
UNICEF: United Nations children’s fund
WHO: World Health Organization
WHR: Waist-hip ratio
WHtR: Waist to height ratio
