# Supplementary material for: Nutritional status and Cardiometabolic health among adolescents; findings from southwestern Nigeria
Source: BMC Nutr. 2019 Dec 2;5:45. doi: 10.1186/s40795-019-0308-5 (PMC7050742; doi:10.1186/s40795-019-0308-5)
Supplement: Supplementary file 1 — Questionnaire. (DOCX 20 kb) [file 40795_2019_308_MOESM1_ESM.docx]

**ADDITIONAL FILE 1**

**QUESTIONNAIRE**

**Description:** Microsoft document highlighting questions/measurements taken from respondents

Participant Identification Number └─┴─┴─┘

**Section A: Socio-Demographic Data of the Respondents**

1. Age in years (as at the last birthday) ____________________________

2. Gender a) Male [ ] b) Female [ ]

3. Religion a) Christianity [ ] b) Islam [ ]

c) Traditionalist [ ] d) Others (Specify)_________________

4. Ethnicity a) Yoruba [ ] b) Hausa/Fulani [ ]

c) Igbo [ ] d) Others (Specify)_________________

5. Class a) JSS 1 [ ] b) JSS 2 [ ] c) JSS 3 [ ]

d) SSS 1 [ ] e) SSS 2 [ ] f) SSS 3 [ ]

6. Number of Children in the family _____________________

7. Birth order a) First [ ] b) Second [ ] c) Third [ ]

d) Fourth [ ] e) Greater than 4 [ ]

8. Father’s occupation

a) Unemployed/ Retired [ ]

b) Skilled worker (eg Professionals (Doctor, lawyers, engineers etc) [ ]

c) Semi-skilled (Artisans, traders) [ ]

c) Unskilled worker (eg labourers, cleaners etc) [ ]

9. Mother’s occupation

a) Unemployed/ Retired [ ]

b) Skilled worker (e.g. Professionals (Doctor, lawyers, engineers etc.) [ ]

c) Semi-skilled (Artisans, traders) [ ]

c) Unskilled worker (e.g. labourers, cleaners etc.) [ ]

10. Family setting a) Monogamous [ ] b) Polygamous [ ]

11. Custodian a) Single parent; Divorced [ ] b) Single parent; Widowed/Widower [ ]

c) Both parents [ ] c) Guardian; Relatives [ ] d) Guardian; Non-relatives [ ]

**Section B: Risk Factors among the Respondents**

12. Have you smoked in any form before? a) Yes [ ] b) No [ ]

13. Do you currently smoke a) Yes [ ] b) No [ ]

14. Have you ever taken alcohol before? a) Yes [ ] b) No [ ]

15. Do you currently take alcohol? a) Yes [ ] b) No [ ]

16. Any history of Hypertension in your Father? a) Yes [ ] b) No [ ] c) I don’t know [ ]

17. Any history of Hypertension in your Mother? a) Yes [ ] b) No [ ] c) I don’t know [ ]

18. Any history of Diabetes in your Father? a) Yes [ ] b) No [ ] c) I don’t know [ ]

19. Any history of Diabetes in your Mother? a) Yes [ ] b) No [ ] c) I don’t know [ ]

**Section C: Anthropometric, Blood Pressure and Blood Sugar Measurements**

20. Weight (in kg) ___________________________________________

21. Height (in meters) _________________________________________

22. Waist circumference (in cm) __________________________________________

23. Hip circumference (in cm) _______________________________________

24. Blood pressure (in mmHg): Systolic_______________ Diastolic____________

25. Random blood glucose level__________(mmol/l)
